# Perturbations of Ubiquitin-Proteasome-Mediated Proteolysis in Aging and Alzheimer’s Disease

**DOI:** 10.3389/fnagi.2019.00324

**Published:** 2019-12-06

**Authors:** Ashok N. Hegde, Spencer G. Smith, Lindsey M. Duke, Allison Pourquoi, Savannah Vaz

**Affiliations:** Department of Biological and Environmental Sciences, Georgia College and State University, Milledgeville, GA, United States

**Keywords:** memory, learning, tau, amyloid beta, protein degradation, cognitive impairment, synaptic plasticity

## Abstract

The ubiquitin-proteasome pathway (UPP) has multiple roles in the normal nervous system, including the development of synaptic connections and synaptic plasticity. Research over the past several years has indicated a role for the UPP in aging without any overt pathology in the brain. In addition, malfunction of the UPP is implicated in Alzheimer’s disease (AD) and dementia associated with it. In this mini review article, we assess the literature on the role of protein degradation by the UPP in aging and in AD with special emphasis on dysregulation of the UPP and its contribution to cognitive decline and impairment.

## Introduction

Ubiquitin has long been known to be associated with pathologies of the brain, including that of Alzheimer’s disease (AD; Ciechanover and Brundin, 2003). Our understanding of the link between ubiquitin-mediated proteolysis and neurodegenerative diseases such as AD, however, has only begun to improve with the elucidation of the mechanistic details of protein degradation. Although proteolysis by the ubiquitin-proteasome pathway (UPP) was originally assumed to operate only on abnormal proteins, research over many years has shown physiological roles for the UPP in various cells, including neurons. In the UPP, ubiquitin attachment to lysine residues in substrate proteins occurs through a series of enzymatic reactions in a highly regulated manner. Additional ubiquitin molecules are attached to the first ubiquitin to form a polyubiquitin chain. The polyubiquitinated protein is degraded by the proteasome (Glickman and Ciechanover, 2002).

In this mini review article, we provide a brief overview of the research on the UPP in the context of normal aging as well as AD and examine the effect on cognition wherever possible.

## UPP and the Aging Brain

Several cellular functions are altered with aging. It is reasonable to hypothesize that ubiquitin-proteasome-mediated proteolysis is also impaired with aging. Investigations, however, have not yielded consistent results. For example, proteolysis by the UPP was tested in mice of different ages using GFP-reporter mice (Cook et al., 2009) that expressed GFP along with a short amino acid sequence (ACKNWFSSLSHFVIHL), originally identified in yeast, that serves as a signal for degradation by the UPP (Gilon et al., 1998). The authors used organotypic hippocampal culture and whole-brain sections and found no significant changes in the amount of GFP from 6 to 18 months of age. It is not clear whether the degradation of the GFP-reporter protein mimics the degradation of endogenous substrates of the UPP. In addition, this study used MG-132, which is not a highly specific proteasome inhibitor (Cook et al., 2009).

Another study tested the capacity of the 26S proteasome to degrade polyubiquitinated substrates in the cortex, hippocampus, and cerebellum of 3-week-old (young) and 24-month (old) rats and found no impairment in degradative capacity with aging. Additionally, despite age-dependent augmentation in overall cellular protein content, there were no increases in the quantity of the proteasome as measured by rocket immunoelectrophoresis (Walker, 1984). Therefore relative to total cellular protein, the percentage of the proteasome was lower in brain regions of the old rats (19% less in cortex, 31% less in the cerebellum, and 37% less in the hippocampus) compared to corresponding regions in the brains of young rats (Giannini et al., 2013). When considered in relation to human aging, the rat model at 24 months is slightly younger compared to the mouse model at 18 months. This is because the average lifespan of rats is 36 months, and that of mice is 24 months, and thus an 18-month-old mouse is equivalent to a 60-year-old human whose lifespan is 80 years (Sengupta, 2013; Ackert-Bicknell et al., 2015).

Genetic experiments using *Drosophila*, however, found a decrease in 26S proteasome function with increasing age as measured in whole flies or fly heads. This study was based on the identification of Rpn11 (a subunit of the proteasome) as a suppressor of age-related neurodegeneration. The amount of Rpn11 was decreased at an age where memory impairment is seen in flies (*Drosophila* lifespan is 60–70 days; 20–30 days post eclosion is considered “old age”; Tonoki et al., 2009).

Other investigations tested specific questions with respect to aging, such as susceptibility to intermittent hypoxia. One study tested proteasome activity and CREB phosphorylation along with making behavioral assessments. With intermittent hypoxia, CREB phosphorylation and proteasome activity decreased, and spatial learning was impaired. All these effects were more pronounced in aged rats compared to young (Gozal et al., 2003).

The relationship between the UPP and aging in the nervous system has also been investigated with respect to ubiquitin-conjugating enzymes. For example, a ubiquitin ligase called mahogunin (Mgrn1) is mostly cytoplasmic in hippocampal neurons. With aging, much of Mgrn1 is found in the nucleus, where it associates with transcriptionally active regions to induce expression of genes critical for coping with a reduction in proteolytic activity (Benvegnù et al., 2017).

A molecule critical for synaptic plasticity and thus cognition, Arc, has also been investigated with respect to aging. Arc is controlled at multiple levels, including transcription and ubiquitin-mediated degradation. In aged rats (24 months), the basal level of Arc is increased, and this was thought to be the result of decreased degradation. Consistent with this idea, levels of Ube3a, the ubiquitin ligase that targets Arc for degradation, is decreased in the hippocampus of aged rats (Fletcher et al., 2014).

## The UPP and AD

**Figure 1:**
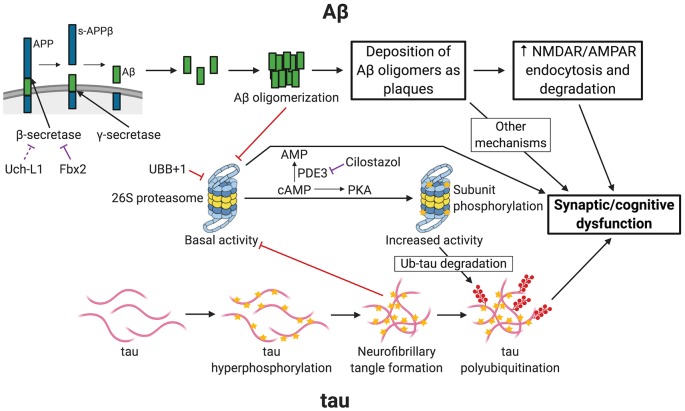
Links between Alzheimer’s disease (AD) and ubiquitin-proteasome-mediated degradation. The figure depicts the pathogenesis of AD and the resultant synaptic/cognitive dysfunction *via* Aβ and tau, the two main postulated mechanisms of AD. The connections between the ubiquitin-proteasome pathway (UPP) and the components of the two pathways are indicated. Only key relevant features of AD pathogenesis are shown. Processes with a purely inhibitory effect are shown by red connectors. Negative regulatory mechanisms with potential for therapeutic intervention are shown by purple connectors. APP, amyloid precursor protein; s-APPβ, soluble N-terminal APP fragment; PDE3, phosphodiesterase 3; PKA, Protein Kinase A (cAMP-dependent protein kinase); Ub-tau, tau with attachment of many ubiquitin (Ub) molecules (i.e., polyubiquitinated tau); stars indicate phosphorylation; shapes with filled red circles with tails represent polyubiquitin attachment. Created with http://biorender.com/.

AD usually affects people 65 years or older, although early-onset familial forms do occur. Much of the patient population falls under the “sporadic” category, in which the exact cause of the disease remains uncertain. AD begins with mild cognitive impairment and, as the disease progresses, patients suffer from severe cognitive defects. At later stages, brain pathologies with plaques and tangles are observed. It is generally accepted that two main types of pathological phenomena occur in the AD brain. One is the accumulation of amyloid β (Aβ), the clumps of which lead to the development of plaques. The second is the accumulation of phosphorylated microtubule-associated protein tau, which ultimately forms tangles. The UPP is linked to both of these pathways of AD pathogenesis (Figure 1).

### Inhibition of the Proteasome in AD

In AD, ubiquitinated proteins accumulate, and it is believed that the proteolytic system in neurons is overwhelmed by aggregating proteins. Based on this logic, investigations were made of the proteasome in both postmortem human AD brains and in the brains of AD model mice. To help understand the results of these experiments, a brief description of the structure of the proteasome is necessary.

The main proteolytic complex that degrades the polyubiquitinated proteins is the 26S proteasome, comprising a cylindrical 20S catalytic core and a 19S regulatory particle (RP) attached on either side of the 20S cylinder. The function of the 19S RP is to recognize and bind the polyubiquitinated substrate proteins, unfold them, and channel them into the narrow opening (13 Å) of the catalytic core for degradation. The catalytic core consists of seven α subunits in two outer rings and seven β subunits in the two inner rings of the cylindrical shape. The 20S core has trypsin-like, chymotrypsin-like, and postglutamyl peptidase activities (Hegde, 2004).

Some of the studies of postmortem AD brains measured the catalytic activities of the 20S proteasome. One study that obtained postmortem brains within ~2.5 h of autopsy found a decrease in chymotrypsin-like and postglutamyl peptidase activities in the parahippocampal gyrus, superior and middle temporal gyri, and inferior parietal lobule. This study found no change in the overall quantities of the α and the β subunits of the 20S proteasome (Keller et al., 2000).

The proteasome is also inhibited in the brains of AD model mice. For example, in mice carrying mutant amyloid precursor protein (APP) transgene (Tg2576), Aβ_(1–42)_ accumulate in neurons, which adversely affects the sorting of membrane receptors [such as the epidermal growth factor receptor (EGFR) and TrkB receptor] through the multivesicular body (MVB). The MVB pathway is critical for the degradation of the endocytosed plasma membrane receptors, which can occur through the lysosome or the proteasome. Investigation of the EGFR, using cortical and hippocampal neuronal cultures from Tg2576 mice and wildtype littermates, found that both ubiquitination and proteasome-mediated degradation of the receptor are impaired in neurons of APP mutant mice. The study also showed that proteasome activity in the neurons of mutant mice was reduced by about 50% (Almeida et al., 2006).

Other studies also support the idea of proteasome inhibition by Aβ_(1–42)_. When cultured neurons were treated with Aβ_(1–42)_, the peptide was found to enter the cells and inhibit proteasome activity as measured by the amount of a GFP-fused reporter construct. The same study also found decreased chymotrypsin-like activity of the proteasome in the hippocampus and cortex of AD model (Tg2576) mice (Oh et al., 2005). An investigation using triple transgenic AD model mice carrying PS1 (M146V), APP (Swe), and tau (P301L) transgenes showed that between 3 and 15 months of age, all three activities of the proteasome decreased in the brains of these mice. Additionally, when pre-pathological hippocampal slices from 3x-Tg mice were maintained in organotypic culture and were treated with the proteasome inhibitor epoxomycin, tau accumulation increased in neurons. Furthermore, the injection of epoxomycin into cerebral ventricles in 4-month-old 3x-Tg-AD mice increased Aβ and tau in the CA1 neurons of the hippocampus, as revealed by immunohistochemistry (Tseng et al., 2008). Earlier studies showed that in the 3x-Tg mice, LTP is reduced at 6 months of age before the development of plaques and tangles. Given that proteasome activity is impaired from 3 months in these mice (Oddo et al., 2003), it is possible that proteasome inhibition contributes to synaptic dysfunction in them.

### Immunoproteasome and AD

The activity of the 20S core of the proteasome can be modulated by substitution of specific subunits. For example, in response to interferon-γ signaling, three subunits, β1, β2, and β5, can be substituted with β1i, β2i, and β5i to enable antigen presentation in the immune system (Hegde, 2004). This type of 20S is called the “immunoproteasome,” which is also found in the brain. A study found an increase in the immunoproteasome in AD brains compared to the brains of the nondemented elderly. Additionally, polymorphisms in a substituted protein (Lmp2 aka β1i) in codon 60 (R/R or R/H) were found. Unaffected brain areas in AD patients with the R/R genotype showed increased proteasome activity compared to corresponding brain areas of AD patients with the R/H genotype (Mishto et al., 2006). Immunoproteasome activity is also increased in reactive glia surrounding plaques in AD model mice (APPswePS1dE9; Orre et al., 2013).

### Enzymes of the UPP and AD

The enzymes of the UPP also interact with the pathological processes mediated by Aβ. For example, expression of a ubiquitin-conjugating enzyme, E2-25K/Hip-2, was upregulated when cultured neurons were exposed to Aβ. In addition, E2-25K/Hip-2 activity was required for proteasome inhibition and for Aβ-mediated neurotoxicity (Song et al., 2003). Later studies found that in AD model mice, E2-25K/Hip-2 stabilizes Caspase-12 by inhibiting the proteasome and the active Caspase-12 mediates ER-stress-induced cell death. Cells that lack E2-25K/Hip-2 are resistant to Aβ-mediated neurotoxicity (Song et al., 2008).

Decrease in a deubiquitinating enzyme, Uch-L1, has been shown to increase the amounts of BACE1 (β-secretase 1), which in turn causes a rise in the levels of Aβ, as has been shown with experiments using AD model mice (5xFAD Tg mice; Guglielmotto et al., 2017).

## Link Between the UPP and Tauopathy

Accumulation of neurofibrillary tangles because of hyperphosphorylated tau is a key pathological process in AD as well as in some other diseases of the nervous system such as frontotemporal degeneration and is referred to as “tauopathy.” Several studies have established that tau is a target of the UPP, and deficits in this pathway might play a role in the etiology of AD.

Previous studies found that proteasome activation by rolipram—a phosphodiesterase 4 (PDE4) inhibitor—decreased tau levels and improved cognition. Subsequently, an FDA-approved PDE3 inhibitor called cilostazol was tested on double transgenic rTg4510 mice overexpressing human mutant (P301L) tau. Experimental mice received intraperitoneal injections of cilostazol twice a day for 30 days, which significantly reduced disease-associated phosphorylated tau forms and the insoluble tau forms (Schaler and Myeku, 2018).

How does cilostazol exert its effect? Cilostazol administration increased the hydrolyzing activity of the proteasome, as shown by fluorogenic substrate kinetic analysis. Immunoblots revealed that the cilostazol-mediated increase of proteasome function was due to phosphorylation of serine and threonine residues by protein kinase A on multiple proteasome subunits. The levels of the proteasome subunits did not change (Schaler and Myeku, 2018).

Activation of the proteasome and the decrease in insoluble forms of tau seemed to ameliorate the cognitive impairment associated with tauopathy. When the rTg4510 mice were subjected to spatial learning testing using the Morris Water Maze (MWM), it was found that cilostazol administration significantly improved the cognitive performance as manifested by reduced escape latency in the MWM (Myeku et al., 2016). Another study showed that PKA-mediated phosphorylation of Rpn6 (part of 19S RP) can increase the activity of the proteasome and boost the degradation of misfolded proteins (Lokireddy et al., 2015).

## Association Between Aβ, the UPP, and Glutamate Receptors Critical for Synaptic Plasticity

Studies investigating the possible connection between Aβ and the UPP have focused on the effect of Aβ on surface expression of NMDA and AMPA receptors. Because cognitive impairment precedes overt pathology in AD and Aβ oligomers cause synaptic dysfunction and memory impairment in AD model mice, it was logical to investigate the glutamate receptors critical for synaptic plasticity.

An investigation using cultured cortical neurons from APP_Swe_ mice and wildtype littermates showed decreased surface expression of NR1 NMDA receptors and reduced signaling to CREB. Also, Aβ was found to activate the tyrosine phosphatase STEP. Later studies found a connection between the UPP and internalization of NMDARs *via* tyrosine phosphatase striatal-enriched protein tyrosine phosphatase 61 (STEP61). Enhanced activity of STEP61 causes decreased surface expression of NR1 and NR2B subunits. STEP61 dephosphorylates a critical tyrosine residue (Tyr-1472) in NR2B subunits, leading to increased endocytosis of NR subunits. Normally, ubiquitin-proteasome-mediated degradation of STEP61 keeps the internalization of NR subunits in check. An increase in Aβ increases STEP61 activity and inhibits the degradation of STEP61. Thus, the overall effect is greatly enhanced STEP61 activity and augmented endocytosis of NMDAR, which leads to synaptic deficits (Kurup et al., 2010).

Previous reports have shown that AMPA receptor synaptic accumulation, trafficking, and turnover are altered in animal models of AD and human patients (Guntupalli et al., 2017). A mechanism responsible for the regulation of AMPA receptors is its proteolysis by the UPP. Specifically, AMPA receptor degradation is mediated by the E3 ligase Nedd4 and deubiquitinating enzyme USP46 (Lin et al., 2011; Huo et al., 2015). *Usp46* mutant mice exhibit alterations in cognitive behaviors (Tomida et al., 2009).

In a recent study, researchers exposed cultured mouse cortical and hippocampal neurons to Aβ and silenced Nedd4 with siRNAs. Aβ exposure for 24 h and 48 h elicited an overall reduction in the AMPA GluA1 subunit by 28% and 53%, respectively. Moreover, the expression of this subunit was significantly reduced in the dendrites of hippocampal neurons as a result of increased internalization (Zhang et al., 2018).

## Other Modes of Connections Between the UPP and AD

Impairment in the UPP appears to contribute to AD pathology in ways that are not apparently connected to Aβ- or tau-mediated processes. This is evident in the case of frameshift mutation during the transcription of the ubiquitin-B gene, which results in UBB mRNA with the +1 frame (UBB+1). The UBB+1 protein has 20 extra amino acids at the C-terminus compared to normal ubiquitin. Cells can tolerate low levels of UBB+1 protein, but at high levels, UBB+1 inhibits the proteasome. Dysfunction of the proteasome in UBB+1 transgenic mice results in a proteomic profile that is similar to that of human AD brains and brains of several AD mouse models (Fischer et al., 2009). These mice show spatial memory deficits in a water maze as well as contextual fear conditioning (van Tijn et al., 2011).

## Manipulating the UPP to Ameliorate AD

Improving the function of UPP components should, in principle, ameliorate some of the symptoms of AD (Gong et al., 2016). Because synaptic dysfunction and cognitive impairment are seen early in AD and the UPP has a role in synaptic plasticity and memory, it might be possible to manipulate the UPP to rescue some deficits. One such attempt was made through the administration of Uch-L1 in a mouse model of AD. This was based on the earlier finding that Uch-L1 plays a role in long-term synaptic plasticity (Hegde et al., 1997). In the wildtype mice, Uch-L1 function was required for inducing hippocampal LTP. In AD model mice (APP/PS1), Uch-L1 function was reduced and could be restored by administering exogenous Uch-L1 protein. Exogenous Uch-L1 also restored LTP in APP/PS1 mice. Moreover, intraperitoneal injection of Uch-L1 fused with the transduction domain of HIV Tat protein (to render it membrane permeant and to allow it to cross the blood-brain barrier) improved contextual memory in APP/PS1 mice (Gong et al., 2006). Overexpression of Uch-L1 slows AD progression in APP/PS45 mice, providing additional evidence for the role of Uch-L1 (Zhang et al., 2014).

Another example is enhancing the degradation of a substrate by manipulating the ubiquitin ligase responsible for targeting it for degradation. BACE1 is a key enzyme in the production of Aβ. BACE1 is degraded by the UPP and is mediated by an SCF ligase. The substrate-binding protein in SCF ligases is the F-box protein. An F-box protein called Fbx2 was found to interact with BACE1. Overexpression of the Fbx2 protein through adenovirus-mediated delivery into neurons derived from AD model mice (Tg2576) reduced the expression of BACE1 and decreased Aβ production. When Fbx2 was overexpressed in the hippocampus of the Tg2576 mice at 12–14 months of age, LTP significantly improved relative to controls. Therefore, reducing BACE1 can potentially improve synaptic dysfunction caused by Aβ (Gong et al., 2010).

## Targeting Components of the UPP for Potential Therapeutic Application

Manipulating the components of the UPP might be beneficial in curtailing pathological development of AD at various stages. Generation of Aβ can potentially be reduced by activating the substrate-interacting component of E3 ligase (Fbx2) that degrades BACE1 (Figure 1). This can, in principle, be done by using small molecules. For example, chemicals that can selectively activate Uch-L1 could reduce the generation of Aβ. Administration of chaperones such as C-terminus of Hsc 70-interacting protein (CHIP) complexed with the heat-shock protein Hsc 70 can stimulate degradation of ubiquitinated tau (Petrucelli et al., 2004; Shimura et al., 2004; Sahara et al., 2005). Also, the design of molecules that increase the activity of the 26S proteasome directly or indirectly (for example, by stimulation of its phosphorylation) could help develop drugs that stimulate degradation of polyubiquitinated tau and enable the proteasome to overcome inhibition by Aβ.

## Future Directions

Based on the research thus far, there is no clear-cut relationship between aging and impairment of the proteasome function. When individual molecules are studied (e.g., mahogunin), however, a clearer picture emerges.

In investigating the connections between the UPP and AD, many studies have focused on transgenic mouse models of AD based on the familial form of the human disease. These models were mainly based on the “Aβ hypothesis” of AD (Mullane and Williams, 2019). Many clinical trials aimed at reducing Aβ levels have not yielded any beneficial outcome for patients except for some encouraging results with drugs that target Aβ oligomers (Panza et al., 2019). There is an increasing realization in the field that alternative hypotheses need to be pursued (Morris et al., 2018). Because the link of the UPP to AD is not just through Aβ, it would be worth investigating how the UPP relates to other factors contributing to AD such as insulin resistance and inflammation in the brain. The development of animal models that recapitulate the sporadic form of the disease which affects most AD patients will be conducive to testing the role of the UPP by itself and in relation to other clearance mechanisms such as autophagy. While we await the development of models for sporadic AD, it might be worthwhile to study postmortem brain samples of patients with sporadic AD as well as neurons generated from patient-derived induced pluripotent cells to test alterations in key components of the UPP. Studies along these lines might be fruitful in years to come.

## Author Contributions

AH, SS, LD, AP and SV contributed to the search and assessment of the available literature. AH wrote the manuscript and other authors helped revise the text to the final form.

## Conflict of Interest

The authors declare that the research was conducted in the absence of any commercial or financial relationships that could be construed as a potential conflict of interest.
